# Associations of childhood experiences with event-related potentials in adults with autism spectrum disorder

**DOI:** 10.1038/s41598-020-70409-z

**Published:** 2020-08-10

**Authors:** Kosuke Okazaki, Toyosaku Ota, Manabu Makinodan, Naoko Kishimoto, Kazuhiko Yamamuro, Rio Ishida, Masato Takahashi, Yuka Yasuda, Ryota Hashimoto, Junzo Iida, Toshifumi Kishimoto

**Affiliations:** 1 Department of Psychiatry, Nara Medical University School of Medicine, 840 Shijo-cho, Kashihara, Nara, 634-8522 Japan; 2 Faculty of Nursing, Nara Medical University School of Medicine, Kashihara, Japan; 3 Life Grow Brilliant Mental Clinic, Osaka, Japan; 4 Department of Pathology of Mental Disease, National Institute of Mental Health, National Center of Neurology and Psychiatry, Tokyo, Japan; 5 Molecular Research Center for Children’s Mental Development, United Graduate School of Child Development, Osaka University, Osaka, Japan

**Keywords:** Biomarkers, Diseases, Health care, Medical research, Pathogenesis, Risk factors, Signs and symptoms

## Abstract

Childhood maltreatment is defined as experiencing of physical, emotional and sexual abuse and neglect in childhood. Maltreatment in childhood leads to substantial psychosocial problems later in life in the general population. Individuals with autism spectrum disorder (ASD) have a higher risk of experiencing stressful and traumatic events, such as maltreatment, during childhood. Although childhood maltreatment reportedly leads to psychosocial problems in adults with ASD, the biological associations between childhood experiences and brain function in this population remain understudied. Here, we evaluated the relationships between childhood experiences and event-related potential (ERP) components during the auditory odd-ball task in adults with ASD (N = 21) and typically developed (TD) individuals (N = 22). We found that the higher the severity of sexual abuse, the larger the amplitude of P300 at Fz, Cz, C3, and C4 in individuals with ASD. Conversely, the severity of child maltreatment was associated with P300 latency at Cz and C3 in TD individuals. Moreover, full IQ was significantly associated with the MMN amplitude at Fz, Cz, C3, and C4 in TD individuals. These findings provide the first evidence that ERPs could be used to study the impacts childhood experiences on the brain of individuals with ASD and that childhood sexual abuse has salient impacts on brain function in this population.

## Introduction

Child maltreatment, including physical, emotional, sexual abuse and neglect, leads to negative impacts in the general population. Multiple studies have revealed that child maltreatment is associated with emotional problems and mental illnesses^1–3^. Green et al. showed that child adversities, including physical and sexual abuse, neglect, parental mental illness, substance abuse disorder, criminality, and family violence, were implicated in 44% of all childhood-onset disorders^4^. Similar to childhood-onset disorders, previous studies have shown that experiencing early-life trauma, such as child maltreatment, negatively impacts individuals in adulthood^5,6^. Briere and Runtz^7^ revealed that physical abuse during childhood had substantial impact on aggressive behavior, sexual abuse with maladaptive sexual conduct, and emotional abuse with low self-esteem in adulthood.

Several studies have indicated that the risk of psychopathologic symptoms and behavioral problems may increase through structural and functional brain changes caused by child maltreatment^8–10^. Previous studies using magnetic resonance imaging have revealed low volume in the subregions of the corpus callosum^11^ and reduced functional connectivity of the amygdala with the hippocampus and anterior cingulate and prefrontal cortices^12,13^. In addition, child maltreatment leads to lower levels of oxytocin^14^ and serotonin^15^ in the brain.

Autism spectrum disorder (ASD) is a neurodevelopmental disorder, characterised by impairments in social and communicative functions and the presence of restricted interests and repetitive behaviour^16^. Corbett et al. proposed that individuals with ASD had a short attention span and showed impulsivity and inattention^17^. Moreover, it has been reported that individuals with ASD have increased risk of experiencing stressful and traumatic life events and that children with intellectual and development disabilities had 1.5 to 3 fold higher risk of maltreatment than children without disabilities^18–20^. Bishop-Fitzpatrick et al.^21^ found that adults with ASD had higher risk of experiencing overall stress compared with typically developed (TD) individuals.

Event-related potentials (ERPs) are commonly used as a non- invasive physiological measure of cognitive dysfunction associated with psychiatric disorders such as ASD^22^, attention deficit/hyperactivity disorder (ADHD)^23–26^, and schizophrenia^27–31^. The P300, a late positive waveform that occurs at a latency of approximately 300 ms after an infrequently-presented target stimulus^32^, is known to reflect executive and attentional function, working memory, event categorisation, and attentional resource allocation^33^. Furthermore, mismatch negativity (MMN) is an ERP component characterised by peak amplitudes at frontocentral electrodes and occurs approximately 100–200 ms after the onset of a deviant stimulus^34^. MMN is considered to reflect an automatic cerebral discrimination process that is not under attentional control^35^. Previous studies have found that the P300 latency is longer^22^ and its amplitude is lower^36^ in adult patients with ASD than in controls. Several studies using ERPs in ASD have found abnormalities of MMN, although no consensus has been reached^37–39^. Kujala et al. showed that the MMN amplitude is lower and the MMN latency is longer in adult patients with ASD than in controls^40^.

Child maltreatment has been related to impulsivity^15^. A previous study showed that impulsive adolescents had increased severity of depression than did non-impulsive adolescents because impulsive adolescents were more likely to employ inappropriate coping techniques when they experienced maltreatment^41^. Impulsivity caused by child maltreatment has been linked to gambling^42^, substance abuse^43^, and suicidal behavior^44^. Additionally, previous studies reports that child maltreatment was associated with ADHD symptoms^45^. Gonzalez et al. showed that foster placement and emotional abuse was associated with ADHD diagnosis^45^. Therefore, previous studies have focused on the control of impulsivity in children and adolescents for therapeutic purposes^46,47^. Our research group previously showed abnormality in ERPs and associations between hyperactivity and impulsivity symptoms and ERP components in children with ADHD^25^. In addition, previous studies have suggested that traumatic events, such as child maltreatment, impact the P300 and MMN components^48,49^. Han et al.^49^ demonstrated that patients with acute stress disorder caused by sexual abuse had reduced P300 amplitude compared with healthy individuals, and Ge et al.^48^ found that the amplitude of MMN was significantly greater in a group that had experienced traumatic events than in a control group.

No studies have examined which components of child maltreatment are related to ERPs in adults with ASD. Individuals with ASD have ADHD-related symptoms^17^, such as inattention, hyperactivity, and impulsivity, and higher risk of experiencing child maltreatment compared with TD individuals^18–20^. Additionally, ADHD-related symptoms and experiencing child maltreatment may affect brain function^13,49,50^. Therefore, here, we evaluated which factors of ADHD-related symptoms or child maltreatment-related variables affect brain function using ERPs with a sample of adults with ASD and TD individuals. In this study, we hypothesized: (a) that adult patients with ASD would have abnormal ERP components, as measured with an auditory oddball task; and (b) there would be a correlation between ERP components in adulthood and childhood experiences. To test our hypotheses, we measured ERP components during the auditory oddball task between adult patients with ASD and age- and sex- matched control subject. Additionally, we evaluated childhood experiences and ADHD-related symptoms in adulthood in both groups.

## Materials and methods

### Participants

We recruited 22 Japanese students and staff from Nara Medical University as TD individuals (16 men; mean age, 26.31 ± 4.40 years; 6 women; mean age, 24.17 ± 2.48 years). TD individuals had no history of psychiatric, neurological, or developmental disorders, and they completed the Autism-Spectrum Quotient Japanese version and those who scored ≤ 25 were enrolled in this study. We also included 21 Japanese adults with ASD (15 men, mean age, 25.27 ± 4.20 years; 6 women, mean age, 31.17 ± 9.81 years) from the outpatient clinic at the Nara Medical University Department of Psychiatry in Japan (Table 1). All patients met the Diagnostical and Statistical Manual of Mental Disorders, Fifth Revision (DSM-5), criteria^16^ for ASD and were re-evaluated using the Autism Disorder Observation Schedule-2nd Edition (ADOS-2)^51^. The ADOS-2 is a semi-structured assessment performed by an experienced trained psychologist and has demonstrated > 80% coding reliability across all modules. The ADOS-2 is composed of five modules, which are used depending on age and verbal communication level. There are five domains, i.e., Communication, Social interaction, Play, Repetitive Behaviors, and Other Behavior, in all modules. To allow comparison of the severity of autism symptoms across the five modules, calibrated severity scores can be calculated for the total score as well as social affect and restricted and repetitive behaviour scores. Exclusion criteria for both groups included any neurological disorder, head injury, serious medical condition, or history of substance abuse/dependence. The full-scale intelligence quotient (FIQ) of each participant was estimated using the Similarities and Symbol Search subsets of the Wechsler Adult Intelligence Scale Third Edition^52^, and those with FIQ scores below 70 were identified by a trained psychologist and excluded from the study. In addition, we excluded samples that had more than 20% rejected trials of all trials. This study was approved by the Institutional Review Board of Nara Medical University and conducted in accordance with the Declaration of Helsinki. Written informed consent was obtained from all participants prior to participation in the study.

**Table 1:** Participant characteristic and Amplitudes and latencies of P300 and MMN components.

	Control		Patients with ASD		*t*-value	*p*-value
	n = 22		n = 21			
	Mean	SD	Mean	SD		
Sex (male/female)^a^	16/6		15/6		NA	0.92
Age (years)	25.73	4.18	26.95	6.69	− 0.72	0.48
FIQ (WAIS-III)	109.45	11.44	95.67	14.88	3.39	< 0.05
**P300 amplitude (μV)**						
Fz	14.02	11.94	9.88	13.10	1.08	1.00
Cz	12.01	7.12	8.42	8.21	1.53	0.67
Pz	8.86	6.42	5.48	6.69	1.69	0.50
C3	11.43	7.47	7.32	7.66	1.78	0.41
C4	13.17	9.18	6.03	9.24	2.54	0.08
**P300 latency (ms)**						
Fz	333.68	27.33	364.19	42.62	− 2.81	< 0.05
Cz	330.36	24.99	363.67	43.75	− 3.08	< 0.05
Pz	329.27	24.10	362.57	45.37	− 3.03	< 0.05
C3	332.55	24.65	363.00	44.95	− 2.77	< 0.05
C4	333.77	25.73	362.76	43.34	− 2.68	0.05
**MMN amplitude (μV)**						
Fz	− 6.31	3.98	− 3.85	3.28	− 2.22	0.16
Cz	− 5.10	3.27	− 3.37	2.25	− 2.03	0.25
Pz	− 3.04	3.13	− 2.40	1.83	− 0.82	1.00
C3	− 4.44	2.73	− 2.31	2.00	− 2.93	< 0.05
C4	− 4.35	3.00	− 2.85	2.26	− 1.86	0.35
**MMN latency (ms)**						
Fz	151.55	12.75	161.77	18.64	− 2.11	0.21
Cz	152.06	12.80	162.31	18.56	− 2.12	0.20
Pz	152.43	13.14	162.12	18.51	− 1.99	0.27
C3	152.25	12.97	161.65	20.02	− 1.84	0.37
C4	151.97	12.39	161.62	19.51	− 1.94	0.30

*FIQ* full-scale IQ, *WAIS-III* Wechsler Adult Intelligence Scale, Third Edition, *SD* standard deviation, *NA* not applicable, *MMN* mismatch negativity.

^a^The χ^2^ test was used for testing group differences. Otherwise, *t* tests were used.

### Assessment of trauma history

We used the Japanese version of the Child Abuse and Trauma Scale (CATS) to assess trauma history^53^. The CATS is a 38-item instrument that retrospectively evaluates adverse childhood experiences^54^. Each item is measured on a five-point scale ranging from 0 = never to 4 = always and divided into five main factors of adverse childhood experiences including Neglect/Negative Home Atmosphere, sexual abuse, Punishment, emotional abuse, and Other. In addition, the sum of scores provides the total score.

### Assessment of symptoms of inattention, hyperactivity, and impulsivity

The Conners’ Adult ADHD Rating Scale (CAARS) was used to evaluate ADHD-related symptoms such as inattention, hyperactivity, and impulsivity in the participants. The CAARS is a 66-item instrument that measures the severity of ADHD symptoms. Each item is measured on a four-point scale ranging from 0 (not at all, never) to 3 (very much, very frequently) and divided into four main factors including Inattention/Memory Problems, Hyperactivity/Restlessness, Impulsivity/Emotional Lability, and Problems with Self-concept. In addition, the scale also measures an ADHD Index and three DSM-IV ADHD symptom subscales including Inattentive Symptoms, Hyperactive/Impulsive Symptoms, and ADHD Symptoms Total, and the total score of the raw scores of the eight subscales.

### ERP tasks

Following the guidelines for ERP measurements, we elicited the P300 components with an auditory oddball task^55^ with the NEC Multi Stim II auditory stimulus system (NEC, Tokyo, Japan). Frequent non-target stimuli were presented as 1,000 Hz bursts (P = 0.8), and infrequent target stimuli were presented as 2,000 Hz tone bursts (P = 0.2). Both stimuli were presented with 10-ms rise/fall times for 50 ms at 80-dB intensities and at 1.5-s intervals. Frequent and Infrequent stimuli were randomly given through headphones. Participants held their eyes open to listen for the target stimuli and to show the response by pushing a button as quickly as possible when hearing target stimuli. We used the same system to elicit the MMN components. Standard stimuli were presented as 1,000 Hz tone bursts (P = 0.9), and deviant stimuli were presented as 1,100 Hz bursts (P = 0.1), which were presented for 50 ms at 80-dB intensities and at 500-ms intervals. The frequent and infrequent stimuli were randomly presented through headphones. We measured the MMN components while participants read books or magazines of their own choice without paying specific attention to the auditory stimuli.

### Recording and analysis

ERPs were recorded using an MEB 2200 evoked potential measuring system (Nihon Kohden, Tokyo, Japan). Electroencephalography (EEG) was recorded at the Fz, Cz, Pz, C3, and C4 positions on the scalp with disk electrodes. All electrodes were re-referenced offline to the average of two mastoid electrodes. The impedance of electrodes was set at ≤ 5 kΩ. The continuous Artifact-free responses to the stimuli were added and then averaged after excluding trials with EEG amplitudes ≥ 100 μV. A digital bandpass filter set from 0.5 to 70 Hz (attenuation by 12 dB/octave). Trials with artifacts caused by muscular activity and complex eye movements were excluded through initial visual inspection of the data by an experienced researcher. Data were corrected for eye-movement artifacts^55,56^. The continuous EEG was segmented into epochs starting 160 ms and ending 640 ms after the stimulus in P300 tasks, and starting 120 ms and ending 400 ms after the stimulus in MMN tasks. The amplitude was measured with the potential of 0 ms latency as the baseline. Each trial was conducted only once to prevent participant fatigue. Finally, we excluded samples that had more than 20% rejected trials of all trials.

#### P300

The auditory oddball task was performed for 240 s. Frequent and infrequent stimuli were given 120 and 30 times, respectively, and the sample rate was 1,000 Hz. Thirty responses to infrequent stimuli were averaged, and P300 was identified as a negative wave with a peak latency occurring between 250 and 550 ms. The mean latency and amplitude were calculated.

#### MMN

The auditory oddball task was performed for 250 s. Frequent and infrequent stimuli were presented 450 and 50 times at 5,000 Hz. The 450 responses to the frequent standard stimuli and the 50 responses to the infrequent deviant stimuli were averaged, and a waveform was calculated as the difference between the averaged waveforms (frequent minus infrequent). The MMN was estimated as a negative wave from the difference waveform with a peak latency between 100 and 250 ms, and the latency and amplitude were measued.

### Statistical analyses

We used PASW Statistics 18.0J for Windows (SPSS, Tokyo, Japan) for the statistical analyses. We conducted a statistical comparison of participant characteristics for each group using two-tailed paired *t* tests. We compared the latencies and amplitudes of the P300 and MMN components, CATS scores, and CAARS scores between the control group and the ASD group using Student’s *t* test. In addition, we have conducted the Analyses of Covariance (ANCOVA) to control the effect of FIQ on ERP components. Spearman’s correlation coefficients (rho) were calculated for the relationships between CATS scores and CAARS scores and between ERP components and CAARS scores in the two groups. In addition, multiple linear stepwise regression analysis was performed to determine the independent factors influencing the ERP components separately for the control and ASD groups. The independent variables used were six factors of the CATS, including Neglect/Negative Home Atmosphere, sexual abuse, Punishment, emotional abuse, Other, and the total score, nine factors of the CAARS, including Inattention/Memory Problems, Hyperactivity/Restlessness, Impulsivity/Emotional Lability, Problems with Self-concept, ADHD Index, Inattentive Symptoms, Hyperactive/Impulsive Symptoms, ADHD Symptoms Total, and the total score of the raw scores of the eight subscales in CAARS, as well as FIQ. Bonferroni correction was conducted to adjust the results of all analyses. Bonferroni-adjusted P-values are reported. P-values 0.05 were considered statistically significant.

## Results

### Demographic data

Demographic characteristics are presented in Table 1. The participant groups did not differ in terms of sex (χ^2^ = 0.009, *df* = 1, P = 0.92) or age (*t* = − 0.72, *df* = 41, *p* = 0.48). While they differed in terms of average FIQ (*t* = 3.39, *df* = 41, *p* = 0.001).

### Comparison of P300- and MMN-component characteristics between patients with ASD and TD individuals

**Figure 1:**
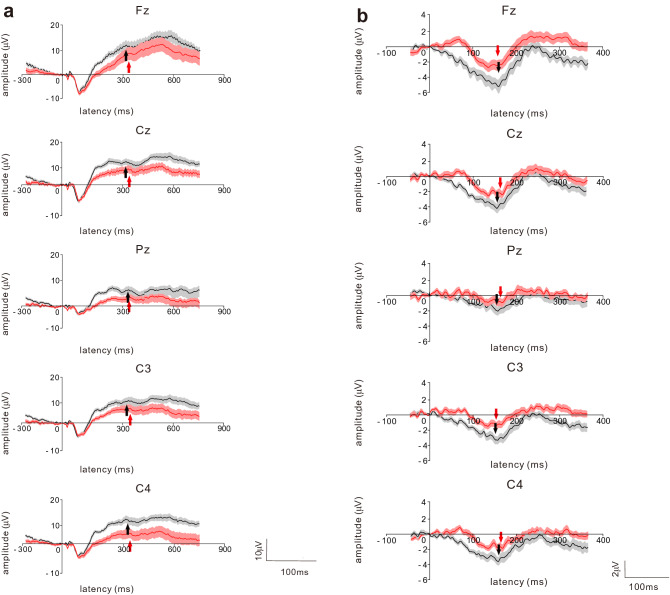
Grand average P300 component and mismatch negativity (MMN) in typically developing (TD) individuals and patients with autism spectrum disorder (ASD).

We show the individual average waveforms in the Supplementary Materials (see Figure S1–S4). We found that the grand average of the P300 latencies at Fz, Cz, Pz and C3 of patients with ASD were longer than those of TD individuals (Fz: *t* = − 2.81, *df* = 41; *p* < 0.05) (Cz: *t* = − 3.08, *df* = 41.00; *p* < 0.05) (Pz: *t* = − 3.03, *df* = 41; *p* < 0.05) (C3: *t* = − 2.77, *df* = 41; *p* < 0.05) (Table 1 and Fig. 1a). We also found that the grand average of the MMN amplitude at C3 in patients with ASD was smaller than that in TD individuals (*t* = − 2.93, *df* = 38.45; *p* < 0.05) (Table 1 and Fig. 1b). Although we have conducted to control the effect of FIQ on ERP components, we found that FIQ have no associations, as covariate, with each ERP components.

### Comparison of CATS scores between patients with ASD and TD individuals

We found that the scores on several CATS subscales were higher in patients with ASD than in controls. The total score of CATS in the ASD group was higher than that in the TD group (*t* = − 3.26, *df* = 41; *p* < 0.05). Further, patients with ASD had higher sexual abuse scores compared with TD individuals (*t* = − 2.88, *df* = 41; *p* < 0.05), and Neglect/Negative Home Atmosphere (*t* = − 3.20, *df* = 41; *p* < 0.05) and emotional abuse (*t* = − 2.77, *df* = 41; *p* < 0.05) in the ASD group were higher than those in the control group (Supplementary Table S1).

### Comparison of CAARS scores between patients with ASD and TD individuals

We evaluated ADHD-related symptoms such as inattention, hyperactivity, and impulsivity with the CAARS. All subscale scores were significantly higher in the ASD group than in the TD group (The total score of the raw scores of the eight subscales in CAARS (*t* = − 5.51, *df* = 41; *p* < 0.05), Inattention/Memory Problems (*t* = − 5.28, *df* = 41; *p* < 0.05), Hyperactivity/Restlessness (*t* = − 3.01, *df* = 41; *p* < 0.05), Impulsivity/Emotional Lability (*t* = − 4.57, *df* = 41; *p* < 0.05), Problems with Self-concept (*t* = − 5.26, *df* = 36.93; *p* < 0.05), ADHD Index (*t* = − 6.07, *df* = 41; *p* < 0.05), Inattentive Symptoms (*t* = − 3.62, *df* = 41; *p* < 0.05), Hyperactive/Impulsive Symptoms (*t* = − 5.62, *df* = 41; *p* < 0.05), and ADHD Symptoms Total (*t* = − 6.63, *df* = 41; *p* < 0.05) (Supplementary Table S1).

### Correlation between ERP components and ADHD symptoms

Our group previously showed that MMN reflects the severity of ADHD symptoms in children and adolescents with ADHD^25^. Therefore, we evaluated the relationships between ERP components and ADHD symptoms in adult patients with ASD and TD individuals. Unexpectedly, in contrast to the previous findings regarding children and adolescents with ADHD, we found no correlations between ERP components and ADHD symptoms in either group (Supplementary Table S2).

### Correlation between adverse childhood experiences and ADHD symptoms

The Spearman’s rho correlations between the six scores in the CATS and nine scores in the CAARS are shown in Supplementary Table S3. In the ASD group, there were positive correlations between the CATS and CAARS scores, except for Punishment (Supplementary Table S3). In contrast, there was no correlation between the CATS and CAARS scores in the TD group (Supplementary Table S3).

### Multiple linear stepwise regression analysis of factors associated with the ERP components in patients with ASD and TD individuals

We found significant correlations between child maltreatment and ADHD-related symptoms in adulthood in the ASD group. Therefore, we conducted multiple linear stepwise regression analysis to determine the factors affecting the ERP components in adulthood. In the ASD group, sexual abuse was significantly associated with the P300 amplitude at Fz, Cz, Pz, C3, and C4. Similarly, Problems with Self-concept was significantly associated with the P300 amplitude at Cz and C3. In addition, Hyperactivity/Restlessness was significantly associated with the P300 amplitude at Fz. No factors were found to influence MMN amplitude and latency (Table 2). In the TD group, Problems with Self-concept was associated with the P300 amplitude at Pz. The the total score of CATS was associated with P300 latency at Cz, C3, and C4. Similarly, there were correlations between Other scores and P300 latency at Pz. FIQ was significantly associated with the MMN amplitude at Fz, Cz, C3, and C4. In contrast, we found no factors influencing MMN latency (Table 2).

**Table 2:** Multiple linear stepwise regression analysis of factors associated with the ERP components in patients with ASD and TD individuals.

Dependent variable and covariate	B	SE	β	*t*-value	*p*-value
**Control**					
**P300 amplitude**					
**Pz**					
Problems with Self-concept	− 0.385	0.169	− 0.455	− 2.284	< 0.05
**P300 latency**					
**Cz**					
The total score of CATS	− 0.933	0.415	− 0.449	− 2.249	< 0.05
**Pz**					
Other	− 3.655	1.503	− 0.478	− 2.432	< 0.05
**C3**					
The total score of CATS	− 0.913	0.410	− 0.445	− 2.225	< 0.05
**C4**					
The total score of CATS	− 1.001	0.422	− 0.468	− 2.369	< 0.05
**MMN amplitude**					
**Fz**					
IQ	− 0.179	0.067	− 0.514	− 2.682	< 0.05
**Cz**					
IQ	− 0.130	0.057	− 0.454	− 2.276	< 0.05
**C3**					
IQ	− 0.107	0.048	− 0.447	− 2.238	< 0.05
C4					
IQ	− 0.132	0.051	− 0.502	− 2.596	< 0.05
**MMN latency**					
NA					
**ASD**					
**P300 amplitude**					
**Fz**					
Sexual abuse	5.194	1.333	0.708	3.895	< 0.01
Hyperactivity/restlessness	− 0.423	0.178	− 0.423	− 2.374	< 0.05
**Cz**					
Sexual abuse	3.909	0.878	0.850	4.452	< 0.01
Problems with self-concept	− 0.387	0.154	− 0.480	− 2.514	< 0.05
**Pz**					
Sexual abuse	1.731	0.762	0.462	2.272	< 0.05
**C3**					
Sexual abuse	3.369	0.875	0.786	3.852	< 0.01
Problems with self-concept	− 0.387	0.153	− 0.514	− 2.522	< 0.05
**C4**					
Sexual abuse	2.770	1.002	0.535	2.764	< 0.05
**P300 latency**					
NA					
**MMN amplitude**					
NA					
**MMN latency**					
NA					

*B* regression coefficient, *SE* standard error, *β* standardized coefficient; these independent factors included FIQ, six factors of CATS including six factors of CATS including Neglect/Negative Home Atmosphere, sexual abuse, punishment, emotional abuse, other and the total score of CATS, and nine factors of CAARS including Inattention/Memory Problems, Hyperactivity/Restlessness, Impulsivity/Emotional Lability, Problems with Self-concept, the ADHD Index, Inattentive Symptoms, Hyperactive/Impulsive Symptoms, and ADHD Symptoms Total, The total score of the raw scores of the eight subscales in CAARS. β coefficient displays strength or influence of the independent variable over the dependent variable including P300 amplitude, P300 latency, MMN amplitude, MMN latency.

## Discussion

To the best of our knowledge, this is the first study to investigate the relationships between child maltreatment and ERP components in adult patients with ASD. We found robust relationships between sexual abuse and P300 amplitudes only in the ASD group. However, there were no relationships between Neglect/Negative Home Atmosphere, Punishment, emotional abuse, or Other scores and ERPs. In contrast, the total score of CATS and Other scores were associated with P300 latency in TD individuals. In addition, FIQ was significantly associated with MMN amplitude at multiple channels only in the TD group.

This study showed that individuals with ASD had prolonged P300 latencies at Fz, Cz, Pz and C3 and reduced MMN amplitude at C3 compared with TD individuals. Consistent with the results of previous studies, we observed prolonged P300 latencies^22^. P300 latency is considered to be related to the interval between perception and reaction and to the monitoring process. Prolonged P300 latencies are associated with cognitive impairment in several psychiatric disorders such as schizophrenia^57,58^ and Alzheimer’s disease^59^. Therefore, we suggest that adults with ASD have impairments in perception and reaction and monitoring process. Similarly, there has been no MMN study with adults with ASD. Our present study revealed that the MMN amplitude at Cz was smaller in the ASD group than in the control group. Previous studies showed that MMN was associated with an automatic cerebral discrimination process and shifts in attention. Therefore, it is considered that these abnormalities of ERP components in patients with ASD were associated with the characteristics of ASD, as impairments in social and communicative functioning, and sustained and selective attention.

We showed that individuals with ASD experience maltreatment in childhood to a greater extent compared to healthy individuals. Consistent with our present study, previous studies have found that individuals with ASD are at greater risk of experiencing child maltreatment^18–20^. This study revealed that adults with ASD have more pronounced ADHD-related symptoms such as hyperactivity, impulsivity, and inattention. Furthermore, there were positive correlations between childhood maltreatment and ADHD-related symptoms in the ASD group. Previous studies have also shown that childhood maltreatment affects ADHD-related symptoms in adulthood^15,60^. Conversely, there was no correlation between childhood maltreatment and ADHD-related symptoms in the control group. The difference in the results regarding the relationship between childhood maltreatment and ADHD-related symptoms in adulthood between the ASD and control groups might have been affected by the vulnerability of individuals with ASD to trauma compared to TD individuals^61^.

In the present study, we found no associations between ERP components and CAARS scores in either group. Several studies have suggested that there are correlations between ADHD symptoms and ERP components in both children and adults with ADHD^25,50^. Yamamuro et al.^25^ showed that MMN latency at Pz was positively correlated with ADHD hyperactivity-impulsivity subscale scores, and MMN amplitudes at Pz were negatively correlated with ADHD full-scale, inattention subscale scores and hyperactivity-impulsivity. Therefore, we have examined effects on ERP components by behavioral variables, as ADHD-related symptoms. In contrast to these previous studies, this study showed no correlation between ADHD-related symptoms and ERP components. This difference in results may be related to differences in the tasks and subjects among studies. Previous studies shows the associations between ERP components and age. P300 latency was shown to be shortest at 15 years old, and to be prolonger as subjects grows older^62^. Goodin et al.^62^. found negative correlation between P300 amplitude and age. In addition, latency and amplitude in ERP were effected by differences of difficulty and types of the tasks^63,64^.

Moreover, we found several correlations between ERP components and child maltreatment. In the ASD group, we revealed that sexual abuse in childhood affected the P300 amplitudes in adulthood. P300 amplitude was shown to reflect cognitive function, such as the amount of attentional resources allocated to the stimulus^65^. Therefore, we suggest that the experience of child sexual abuse may affect the amount of attentional resources allocated to the stimulus in adulthood. Several studies have reported a relationship between the ERP components and sexual abuse. Han et al. reported that the P300 amplitudes, which represent neural activity associated with working memory processes and attention^33^, were lower in individuals who had experienced sexual abuse than in control individuals^49^. The disagreement between the previous and present study findings might be attributed to the difference in the profile of the participants, such as in examined disorders and sex.

In the control group, we could not find specific types of abuse impacting brain function in adulthood. Previous studies have shown that individuals who experience a greater degree of adversity in childhood are at higher risk of mental health problems in adulthood^66,67^. Therefore, in the general population, the severity of abuse may affect brain function irrespective of the type of abuse.

Here, we showed that the impact of experiencing child maltreatment on brain function in adulthood differs between TD individuals and those with ASD. In addition, it is also suggested that there were differences between TD individuals and those with ASD in how the types of child abuse affect brain function in adulthood.

There are some limitations that should be considered when interpreting the current findings. First, our sample size was relatively small. Therefore, the small number of samples led a lack of statistical power. Future research with larger samples is required. Second, there was a difference in FIQ between the ASD and control groups; matching the two groups for FIQ would reduce the impact of this variable on the results. Third, the CATS retrospectively evaluates adverse childhood experiences; therefore, we need to consider the effects of recall bias when considering the results. Fourth, we evaluated symptoms such as hyperactivity, impulsivity, and inattention in adulthood solely via the CAARS. Future studies using other neuropsychological test batteries would be useful for validating the present findings. Fifth, no participant in the control group had experienced sexual abuse. Therefore, it was not possible to compare the impact of sexual abuse between TD individuals and those with ASD. Future studies need to consider the impact of sexual abuse in the general population.

## Conclusion

This study showed that sexual abuse in childhood was associated with brain function evaluated by ERP in adulthood in individuals with ASD; therefore, attention on the association with sexual abuse experienced in childhood is needed in this population. Furthermore, we suggest that there were differences between TD individuals and those with ASD in how the types of child abuse correlate brain function in adulthood.

## Supplementary information

Supplementary Figure 1.

Supplementary Figure 2.

Supplementary Figure 3.

Supplementary Figure 4.

Supplementary Table 1.

Supplementary Table 2.

Supplementary Table 3.

## Data Availability

Data are available upon request.

## References

[1] McIntosh JL (1994). Generational analyses of suicide: baby boomers and 13ers. Suicide Life Threat. Behav..

[2] Boney-McCoy S, Finkelhor D (1995). Psychosocial sequelae of violent victimization in a national youth sample. J. Consult. Clin. Psychol..

[3] Kessler RC, Borges G, Walters EE (1999). Prevalence of and risk factors for lifetime suicide attempts in the National Comorbidity Survey. Arch. Gen. Psychiatry.

[4] Green JG (2010). Childhood adversities and adult psychiatric disorders in the national comorbidity survey replication I: associations with first onset of DSM-IV disorders. Arch. Gen. Psychiatry.

[5] Pirkola S (2005). Childhood adversities as risk factors for adult mental disorders: results from the Health 2000 study. Soc. Psychiatry Psychiatr. Epidemiol..

[6] Pechtel P, Pizzagalli DA (2011). Effects of early life stress on cognitive and affective function: an integrated review of human literature. Psychopharmacology.

[7] Briere J, Runtz M (1988). Multivariate correlates of childhood psychological and physical maltreatment among university women. Child Abuse Negl..

[8] De Bellis MD (2002). Brain structures in pediatric maltreatment-related posttraumatic stress disorder: a sociodemographically matched study. Biol. Psychiatry.

[9] Heim C, Shugart M, Craighead WE, Nemeroff CB (2010). Neurobiological and psychiatric consequences of child abuse and neglect. Dev. Psychobiol..

[10] Jensen SK (2015). Effect of early adversity and childhood internalizing symptoms on brain structure in young men. JAMA Pediatr..

[11] Teicher MH, Samson JA, Anderson CM, Ohashi K (2016). The effects of childhood maltreatment on brain structure, function and connectivity. Nat. Rev. Neurosci..

[12] Herringa RJ (2013). Childhood maltreatment is associated with altered fear circuitry and increased internalizing symptoms by late adolescence. Proc. Natl. Acad. Sci. U.S.A..

[13] Jedd K (2015). Long-term consequences of childhood maltreatment: altered amygdala functional connectivity. Dev. Psychopathol..

[14] Heim C (2009). Lower CSF oxytocin concentrations in women with a history of childhood abuse. Mol. Psychiatry.

[15] Braquehais MD, Oquendo MA, Baca-Garcia E, Sher L (2010). Is impulsivity a link between childhood abuse and suicide?. Compr. Psychiatry.

[16] American Psychiatric Association (2013). Diagnostic and Statistical Manual of Mental Disorders.

[17] Corbett BA, Constantine LJ (2006). Autism and attention deficit hyperactivity disorder: assessing attention and response control with the integrated visual and auditory continuous performance test. Child Neuropsychol..

[18] Sullivan PM, Knutson JF (2000). Maltreatment and disabilities: a population-based epidemiological study. Child Abuse Negl..

[19] Hibbard RA, Desch LW (2007). Maltreatment of children with disabilities. Pediatrics.

[20] Reiter S, Bryen DN, Shachar I (2007). Adolescents with intellectual disabilities as victims of abuse. J. Intellect. Disabil..

[21] Bishop-Fitzpatrick L, Mazefsky CA, Minshew NJ, Eack SM (2015). The relationship between stress and social functioning in adults with autism spectrum disorder and without intellectual disability. Autism Res..

[22] Sokhadze E (2009). Event-related potential study of novelty processing abnormalities in autism. Appl. Psychophysiol. Biofeedback.

[23] Barry RJ (2009). Event-related potentials in adults with Attention-Deficit/Hyperactivity Disorder: an investigation using an inter-modal auditory/visual oddball task. Int. J. Psychophysiol..

[24] Sawada M (2010). Effects of osmotic-release methylphenidate in attention-deficit/hyperactivity disorder as measured by event-related potentials. Psychiatry Clin. Neurosci..

[25] Yamamuro K (2016). Associations between the mismatch-negativity component and symptom severity in children and adolescents with attention deficit/hyperactivity disorder. Neuropsychiatr. Dis. Treat..

[26] Barth B (2018). Identification of neurophysiological biotypes in attention deficit hyperactivity disorder. Psychiatry Clin. Neurosci..

[27] Ford JM (1999). P300 amplitude is related to clinical state in severely and moderately ill patients with schizophrenia. Biol. Psychiatry.

[28] Takashima A, Ohta K, Matsushima E, Toru M (2001). The event-related potentials elicited by content and function words during the reading of sentences by patients with schizophrenia. Psychiatry Clin. Neurosci..

[29] Kim MS, Kwon JS, Kang SS, Youn T, Kang KW (2004). Impairment of recognition memory in schizophrenia: event-related potential study using a continuous recognition task. Psychiatry Clin. Neurosci..

[30] Mori K (2012). State and trait markers of emotionally charged visual event-related potentials (P300) in drug-naive schizophrenia. Psychiatry Clin. Neurosci..

[31] Spironelli C, Romeo Z, Maffei A, Angrilli A (2019). Comparison of automatic visual attention in schizophrenia, bipolar disorder, and major depression: evidence from P1 event-related component. Psychiatry Clin. Neurosci..

[32] Halgren E (1980). Endogenous potentials generated in the human hippocampal formation and amygdala by infrequent events. Science.

[33] Polich J (2007). Updating P300: an integrative theory of P3a and P3b. Clin Neurophysiol.

[34] Naatanen R, Paavilainen P, Rinne T, Alho K (2007). The mismatch negativity (MMN) in basic research of central auditory processing: a review. Clin. Neurophysiol..

[35] Jonkman LM (1997). Event-related potentials and performance of attention-deficit hyperactivity disorder: children and normal controls in auditory and visual selective attention tasks. Biol. Psychiatry.

[36] Cygan HB, Tacikowski P, Ostaszewski P, Chojnicka I, Nowicka A (2014). Neural correlates of own name and own face detection in autism spectrum disorder. PLoS ONE.

[37] Gomot M, Giard MH, Adrien JL, Barthelemy C, Bruneau N (2002). Hypersensitivity to acoustic change in children with autism: electrophysiological evidence of left frontal cortex dysfunctioning. Psychophysiology.

[38] Ferri R (2003). The mismatch negativity and the P3a components of the auditory event-related potentials in autistic low-functioning subjects. Clin. Neurophysiol..

[39] Lepisto T (2005). The discrimination of and orienting to speech and non-speech sounds in children with autism. Brain Res..

[40] Kujala T (2005). Neurophysiological evidence for cortical discrimination impairment of prosody in Asperger syndrome. Neurosci. Lett..

[41] d'Acremont M, Van der Linden M (2007). How is impulsivity related to depression in adolescence? Evidence from a French validation of the cognitive emotion regulation questionnaire. J. Adolesc..

[42] Ledgerwood DM, Petry NM (2006). Posttraumatic stress disorder symptoms in treatment-seeking pathological gamblers. J. Trauma Stress.

[43] Zlotnick C (1997). Trauma, dissociation, impulsivity, and self-mutilation among substance abuse patients. Am. J. Orthopsychiatry.

[44] Roy A (2005). Childhood trauma and impulsivity. Possible relevance to suicidal behavior. Arch. Suicide Res..

[45] Gonzalez RA (2019). Evidence of concurrent and prospective associations between early maltreatment and ADHD through childhood and adolescence. Soc. Psychiatry Psychiatr. Epidemiol..

[46] Diseth TH (2005). Dissociation in children and adolescents as reaction to trauma—an overview of conceptual issues and neurobiological factors. Nord. J. Psychiatry.

[47] Henry J, Sloane M, Black-Pond C (2007). Neurobiology and neurodevelopmental impact of childhood traumatic stress and prenatal alcohol exposure. Lang. Speech Hear. Serv. Sch..

[48] Ge Y, Wu J, Sun X, Zhang K (2011). Enhanced mismatch negativity in adolescents with posttraumatic stress disorder (PTSD). Int. J. Psychophysiol..

[49] Han C (2018). Dysfunctional information processing in individuals with acute exposure to sexual abuse: an ERP study. Medicine (Baltimore).

[50] Kim JS, Kim S, Jung W, Im CH, Lee SH (2016). Auditory evoked potential could reflect emotional sensitivity and impulsivity. Sci. Rep..

[51] Lord C, Rutter M, DiLavore PC, Risi S, Gotham K, Bishop SL (2012). Autism Diagnostic Observation Schedule, 2nd edition (ADOS-2).

[52] Sumiyoshi C, Fujino H, Sumiyoshi T, Yasuda Y, Yamamori H, Ohi K, Fujimoto M, Takeda M, Hashimoto R (2016). Usefulness of the Wechsler Intelligence Scale short form for assessing functional outcomes in patients with schizophrenia. Psychiatry Res..

[53] Tanabe H, Ozawa S, Goto K (2010). Psychometric properties of the Japanese version of the Child Abuse and Trauma Scale (CATS).

[54] Sanders B, Becker-Lausen E (1995). The measurement of psychological maltreatment: early data on the Child Abuse and Trauma Scale. Child Abuse Negl..

[55] Picton TW (2000). Guidelines for using human event-related potentials to study cognition: recording standards and publication criteria. Psychophysiology.

[56] Gratton G, Coles MG, Donchin E (1983). A new method for off-line removal of ocular artifact. Electroencephalogr. Clin. Neurophysiol..

[57] Qiu YQ, Tang YX, Chan RC, Sun XY, He J (2014). P300 aberration in first-episode schizophrenia patients: a meta-analysis. PLoS ONE.

[58] Misic B (2015). Coordinated information generation and mental flexibility: large-scale network disruption in children with autism. Cereb. Cortex.

[59] Jiang S (2015). Using event-related potential P300 as an electrophysiological marker for differential diagnosis and to predict the progression of mild cognitive impairment: a meta-analysis. Neurol. Sci..

[60] Matheson SL (2017). Effects of maltreatment and parental schizophrenia spectrum disorders on early childhood social-emotional functioning: a population record linkage study. Epidemiol. Psychiatr. Sci..

[61] Kerns CM, Newschaffer CJ, Berkowitz SJ (2015). Traumatic childhood events and autism spectrum disorder. J. Autism Dev. Disord..

[62] Goodin SD (1978). Age-related variations in evoked potentials to auditory stimuli in normal human subjects. Electroencephalogr. Clin. Neurophysiol..

[63] Squires NK (1975). Two varieties of long-latency positive waves evoked by unpredictable auditory stimuli in man. Electroencephalogr. Clin. Neurophysiol..

[64] Courchesne E (1975). Stimulus novelty, task relevance and the visual evoked potential in man. Electro-encephalogr. Clin. Neurophysiol..

[65] Johnson R, Barnhardt J, Zhu J (2004). The contribution of executive processes to deceptive responding. Neuropsychologia.

[66] Dube SR (2003). Childhood abuse, neglect, and household dysfunction and the risk of illicit drug use: the adverse childhood experiences study. Pediatrics.

[67] Dube SR, Felitti VJ, Dong M, Giles WH, Anda RF (2003). The impact of adverse childhood experiences on health problems: evidence from four birth cohorts dating back to 1900. Prev. Med..

