# Supplementary figures and images for: Associations of childhood experiences with event-related potentials in adults with autism spectrum disorder
Source: Sci Rep. 2020 Aug 10;10:13447. doi: 10.1038/s41598-020-70409-z (PMC7417533; doi:10.1038/s41598-020-70409-z)

Fz

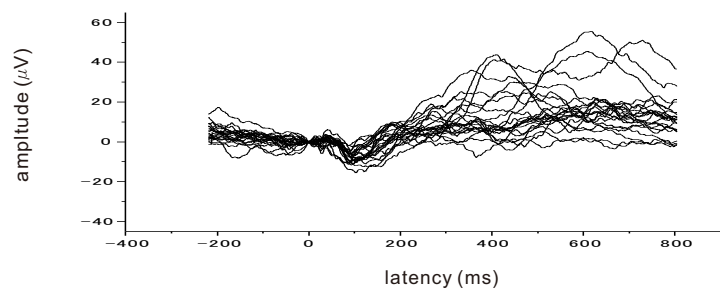

Cz

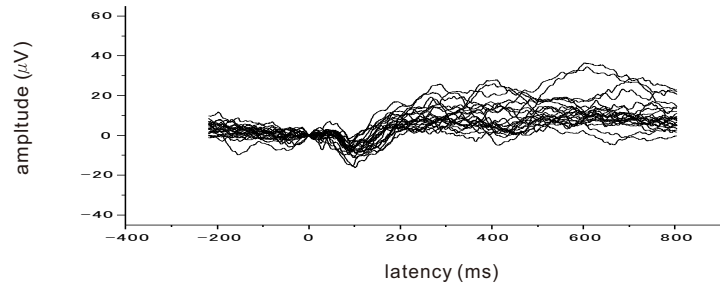

Pz

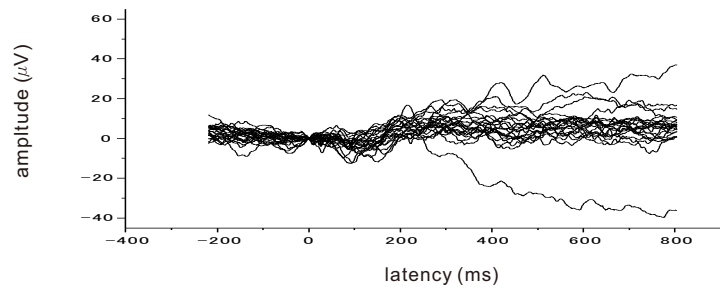

C3

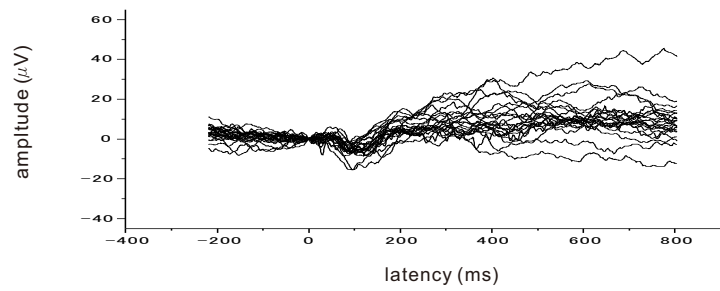

C4

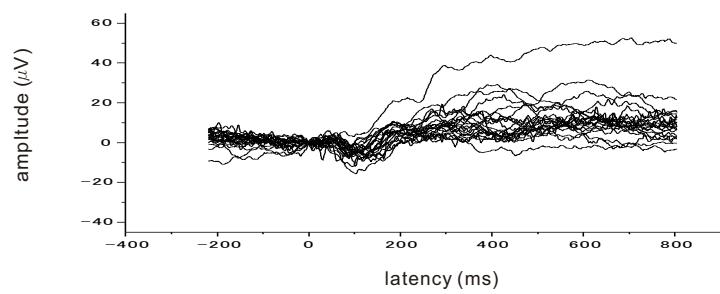

10  $\mu\text{V}$   
100ms

Supplement: Supplementary file 1 — Supplementary Figure 1. [file 41598_2020_70409_MOESM1_ESM.pdf]

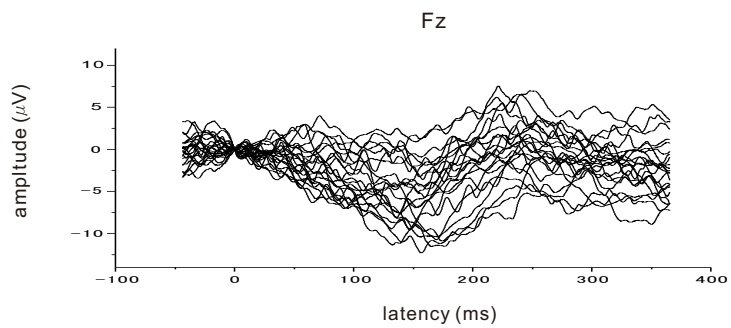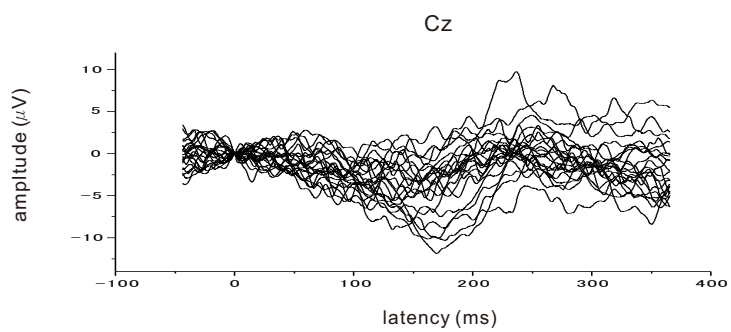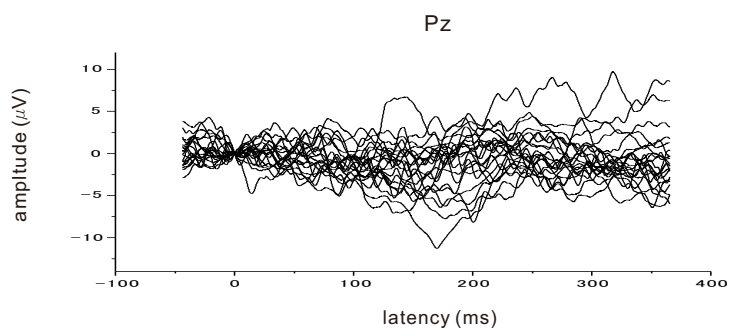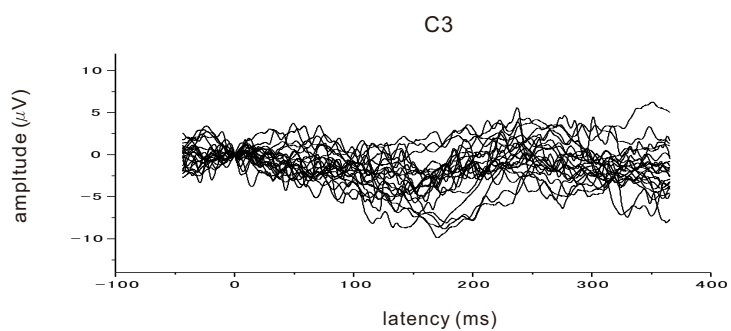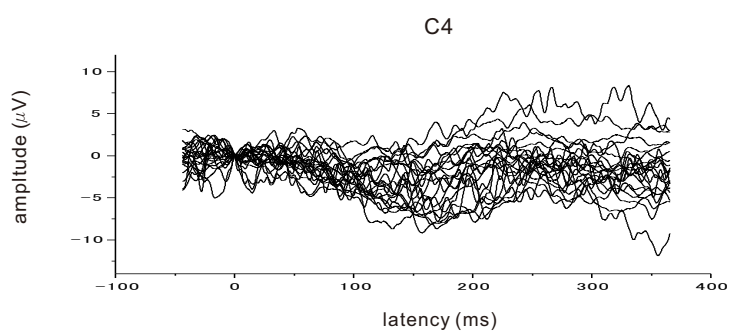

2.5  $\mu\text{V}$   
50ms

Supplement: Supplementary file 2 — Supplementary Figure 2. [file 41598_2020_70409_MOESM2_ESM.pdf]

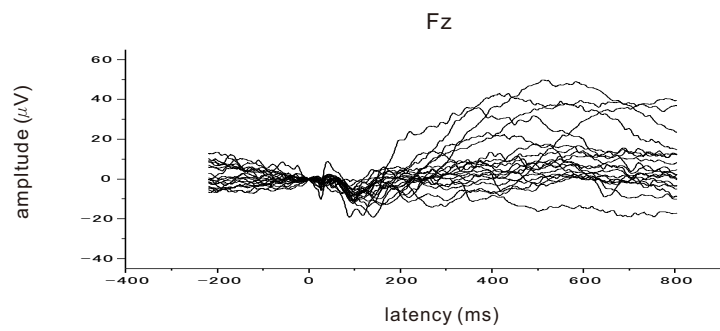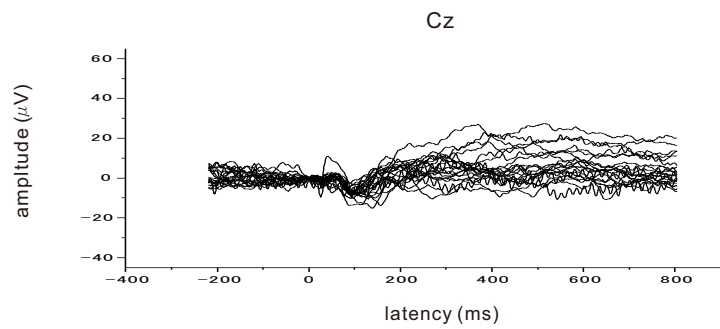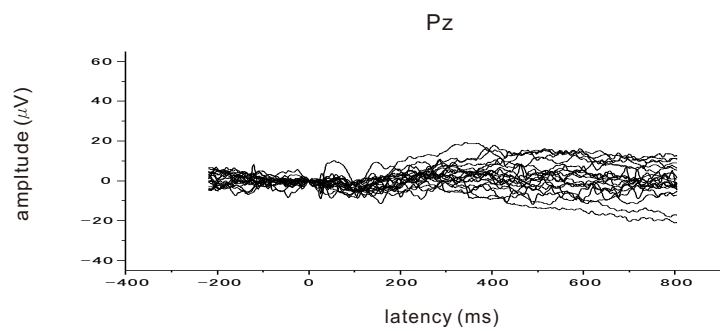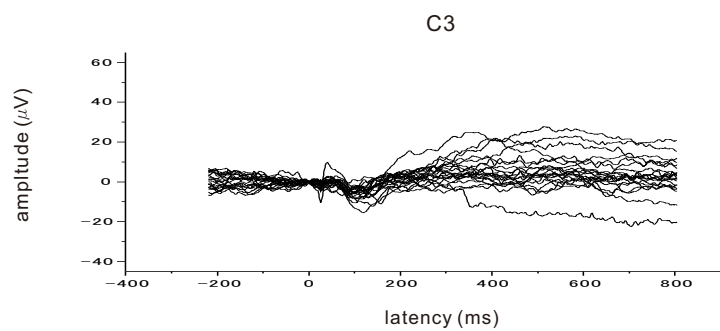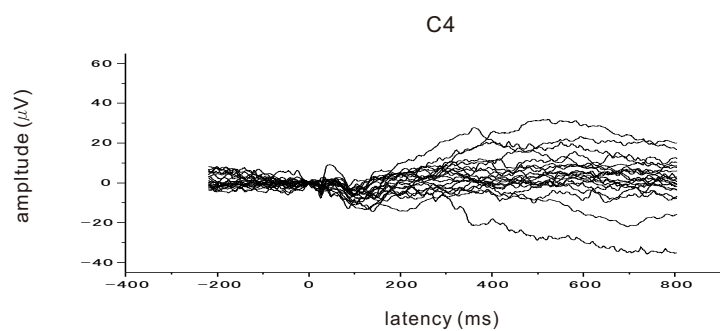

10  $\mu\text{V}$   
100ms

Supplement: Supplementary file 3 — Supplementary Figure 3. [file 41598_2020_70409_MOESM3_ESM.pdf]

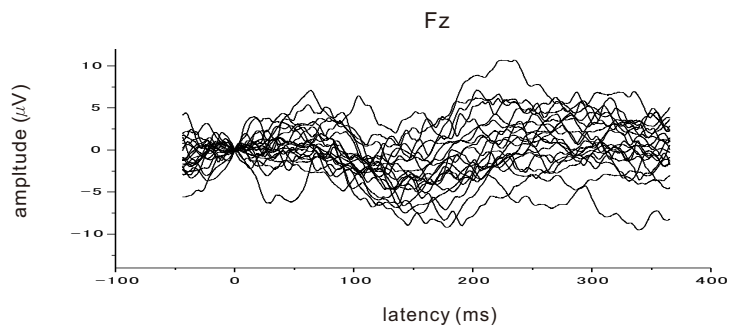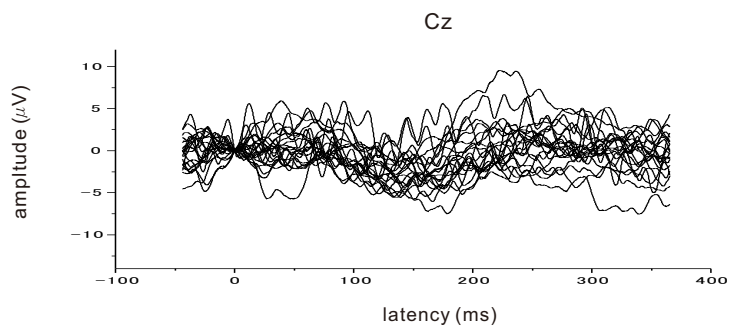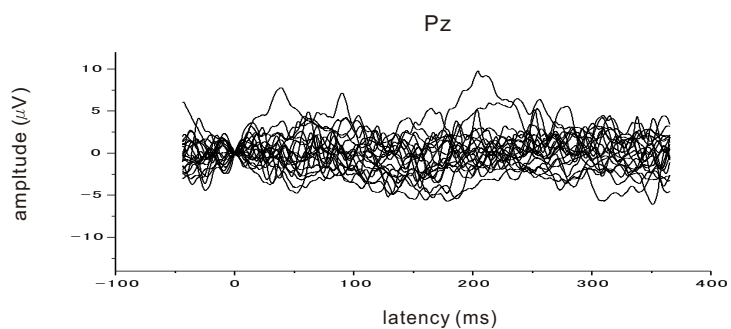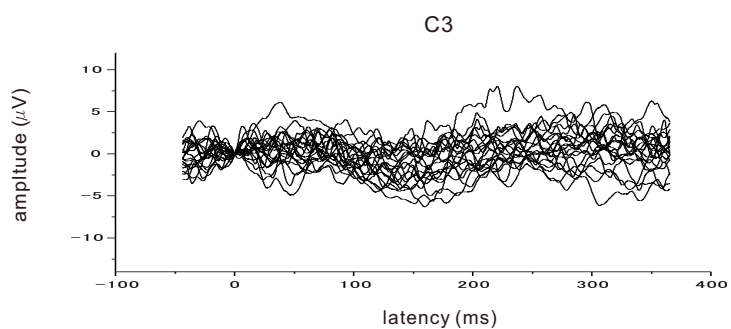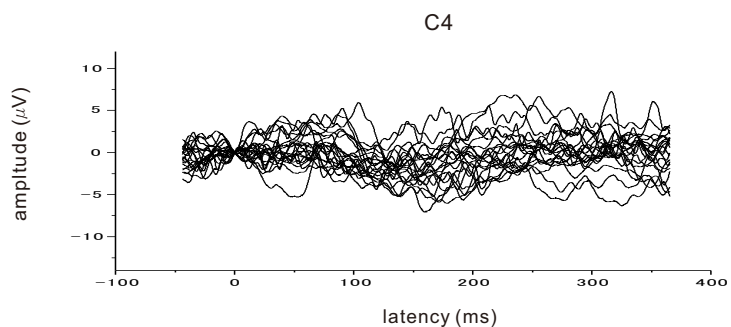

2.5  $\mu\text{V}$   
50ms

Supplement: Supplementary file 4 — Supplementary Figure 4. [file 41598_2020_70409_MOESM4_ESM.pdf]
