# Supplementary material for: Associations of childhood experiences with event-related potentials in adults with autism spectrum disorder
Source: Sci Rep. 2020 Aug 10;10:13447. doi: 10.1038/s41598-020-70409-z (PMC7417533; doi:10.1038/s41598-020-70409-z)
Supplement: Supplementary file 5 — Supplementary Table 1. [file 41598_2020_70409_MOESM5_ESM.docx]

| **eTable 1** |  |  |  |  |  |  |  |  |  |
| --- | --- | --- | --- | --- | --- | --- | --- | --- | --- |
| Comparisons of CATS scores and CAARS scores between control and ASD groups | | | | | | | | | |
|  |  | Control | |  | Patients with ASD | |  |  |  |
|  |  | n=22 |  |  | n=21 |  |  |  |  |
|  |  | Mean | SD |  | Mean | SD |  | *t*-value | *p*-value |
| **CATS scores** |  |  |  |  |  |  |  |  |  |
| CTS |  | 20.23 | 12.04 |  | 42.76 | 29.97 |  | -3.26 | ＜0.05 |
| SA |  | 0.00 | 0.00 |  | 1.10 | 1.79 |  | -2.88 | ＜0.05 |
| Punishment |  | 6.91 | 3.50 |  | 10.05 | 5.16 |  | -2.32 | 0.16 |
| NNHA |  | 6.59 | 4.55 |  | 15.38 | 12.01 |  | -3.20 | ＜0.05 |
| EA |  | 4.45 | 3.35 |  | 10.05 | 8.83 |  | -2.77 | ＜0.05 |
| Other |  | 2.27 | 3.15 |  | 6.19 | 5.99 |  | -2.67 | 0.07 |
| **CAARS scores** |  |  |  |  |  |  |  |  |  |
| TSR |  | 38.55 | 17.21 |  | 92.00 | 41.95 |  | -5.51 | ＜0.05 |
| IMP |  | 45.36 | 6.29 |  | 62.00 | 13.32 |  | -5.28 | ＜0.05 |
| HR |  | 46.14 | 7.23 |  | 55.95 | 13.37 |  | -3.01 | ＜0.05 |
| IEL |  | 43.73 | 6.42 |  | 59.24 | 14.53 |  | -4.57 | ＜0.05 |
| PSC |  | 46.59 | 7.59 |  | 61.05 | 10.19 |  | -5.26 | ＜0.05 |
| AI |  | 46.14 | 6.85 |  | 64.71 | 12.52 |  | -6.07 | ＜0.05 |
| AIS |  | 45.86 | 9.17 |  | 59.14 | 14.45 |  | -3.62 | ＜0.05 |
| AHIS |  | 45.50 | 6.32 |  | 63.57 | 13.63 |  | -5.62 | ＜0.05 |
| AST |  | 43.82 | 6.46 |  | 67.38 | 15.32 |  | -6.63 | ＜0.05 |
| Abbreviations: ASD, autism spectrum disorder; CATS, the Japanese version of the Child Abuse and Trauma Scale; CTS, CATS total score; SA, sexual abuse; NNHA, neglect/negative home atmosphere; EA, emotional abuse. CAARS, the Conners’ Adult ADHD Rating Scale; TSR, CAARS total score of raw scores; IMP, inattention/memory problems; HR, hyperactivity/restlessness; IEL, impulsivity/emotional lability; PSC, problems with self-concept; AI, ADHD Index; ASI, *DSM-Ⅳ* ADHD symptom inattentive Symptoms; ASHI, *DSM-Ⅳ* ADHD symptom hyperactive/Impulsive Symptoms; AST, *DSM-Ⅳ* ADHD symptom ADHD symptoms total | | | | | | | | | |
| t-tests were used. | | | | | | | | | |
