# Supplementary material for: Associations of childhood experiences with event-related potentials in adults with autism spectrum disorder
Source: Sci Rep. 2020 Aug 10;10:13447. doi: 10.1038/s41598-020-70409-z (PMC7417533; doi:10.1038/s41598-020-70409-z)
Supplement: Supplementary file 6 — Supplementary Table 2. [file 41598_2020_70409_MOESM6_ESM.docx]

| **eTable 2** Correlations between ERP components and CAARS scores | | | | | | | | | |
| --- | --- | --- | --- | --- | --- | --- | --- | --- | --- |
|  | Spearman’s correlations | | | | | | | | |
|  | **CAARS scores** | | | | | | | | |
|  | TSR | IMP | HR | IEL | PSC | AI | AIS | AHIS | AST |
| **Control** |  |  |  |  |  |  |  |  |  |
| P300 amplitude |  |  |  |  |  |  |  |  |  |
| Fz | -0.101 | -0.188 | 0.045 | -0.145 | 0.076 | 0.115 | -0.014 | 0.007 | -0.187 |
| Cz | -0.166 | -0.121 | -0.121 | -0.120 | -0.185 | -0.015 | 0.126 | -0.009 | -0.253 |
| Pz | -0.284 | -0.267 | -0.265 | -0.161 | -0.455 | -0.013 | -0.040 | -0.057 | -0.289 |
| C3 | -0.213 | -0.279 | -0.110 | -0.167 | -0.294 | 0.010 | 0.012 | -0.016 | -0.194 |
| C4 | -0.192 | -0.168 | -0.179 | -0.267 | -0.231 | 0.045 | -0.101 | -0.044 | -0.159 |
| P300 latency |  |  |  |  |  |  |  |  |  |
| Fz | -0.079 | -0.190 | 0.230 | -0.121 | -0.197 | -0.170 | -0.200 | -0.016 | -0.139 |
| Cz | 0.040 | -0.174 | 0.368 | 0.010 | -0.063 | 0.202 | -0.107 | 0.066 | -0.115 |
| Pz | 0.050 | -0.164 | 0.402 | 0.050 | -0.056 | 0.205 | -0.086 | 0.073 | -0.148 |
| C3 | 0.007 | -0.171 | 0.324 | 0.018 | -0.149 | 0.215 | -0.167 | 0.039 | -0.101 |
| C4 | 0.016 | -0.144 | 0.262 | -0.016 | -0.156 | 0.183 | -0.139 | 0.039 | -0.121 |
| MMN amplitude |  |  |  |  |  |  |  |  |  |
| Fz | -0.025 | -0.106 | 0.217 | 0.095 | -0.202 | -0.251 | 0.149 | -0.053 | -0.207 |
| Cz | 0.132 | -0.034 | 0.188 | 0.076 | -0.116 | -0.106 | 0.196 | 0.077 | -0.065 |
| Pz | 0.020 | -0.143 | 0.222 | -0.145 | -0.173 | -0.116 | -0.002 | -0.016 | -0.156 |
| C3 | -0.031 | -0.128 | 0.151 | -0.061 | -0.020 | -0.196 | 0.032 | -0.085 | -0.082 |
| C4 | 0.188 | 0.143 | 0.294 | 0.058 | -0.165 | -0.063 | 0.221 | 0.122 | 0.016 |
| MMN latency |  |  |  |  |  |  |  |  |  |
| Fz | 0.015 | -0.063 | -0.145 | 0.063 | -0.126 | 0.084 | 0.203 | 0.222 | 0.032 |
| Cz | 0.048 | -0.019 | -0.139 | 0.045 | -0.122 | 0.121 | 0.182 | 0.249 | 0.084 |
| Pz | 0.039 | -0.020 | -0.168 | 0.032 | -0.129 | 0.116 | 0.178 | 0.240 | 0.081 |
| C3 | 0.036 | -0.017 | -0.162 | 0.020 | -0.125 | 0.128 | 0.168 | 0.246 | 0.084 |
| C4 | 0.027 | -0.052 | -0.141 | 0.058 | -0.132 | 0.119 | 0.160 | 0.235 | 0.093 |
| **ASD** |  |  |  |  |  |  |  |  |  |
| P300 amplitude |  |  |  |  |  |  |  |  |  |
| Fz | -0.144 | -0.175 | -0.090 | 0.006 | -0.064 | -0.214 | -0.021 | -0.114 | -0.075 |
| Cz | -0.157 | -0.242 | -0.151 | -0.044 | -0.012 | -0.212 | -0.021 | -0.135 | -0.046 |
| Pz | -0.071 | -0.200 | 0.016 | -0.066 | 0.040 | 0.046 | -0.036 | 0.007 | -0.018 |
| C3 | -0.203 | -0.371 | -0.167 | -0.045 | -0.129 | -0.203 | -0.119 | -0.167 | -0.180 |
| C4 | 0.036 | -0.133 | 0.071 | 0.045 | 0.071 | 0.029 | 0.047 | 0.059 | 0.044 |
| P300 latency |  |  |  |  |  |  |  |  |  |
| Fz | 0.133 | 0.107 | 0.168 | 0.224 | -0.023 | -0.033 | 0.152 | 0.078 | 0.176 |
| Cz | 0.185 | 0.160 | 0.202 | 0.270 | 0.038 | 0.008 | 0.208 | 0.131 | 0.211 |
| Pz | 0.171 | 0.157 | 0.202 | 0.247 | 0.036 | -0.023 | 0.200 | 0.121 | 0.212 |
| C3 | 0.202 | 0.165 | 0.206 | 0.288 | 0.074 | 0.024 | 0.223 | 0.151 | 0.232 |
| C4 | 0.240 | 0.209 | 0.248 | 0.313 | 0.113 | 0.048 | 0.268 | 0.187 | 0.275 |
| MMN amplitude |  |  |  |  |  |  |  |  |  |
| Fz | 0.143 | 0.245 | 0.193 | -0.140 | 0.280 | 0.268 | 0.036 | 0.194 | 0.199 |
| Cz | 0.038 | 0.072 | -0.010 | -0.128 | 0.100 | 0.080 | -0.087 | 0.025 | 0.033 |
| Pz | -0.044 | -0.118 | -0.206 | -0.044 | 0.056 | -0.116 | -0.032 | -0.075 | -0.018 |
| C3 | 0.183 | 0.163 | 0.096 | 0.016 | 0.332 | 0.160 | 0.167 | 0.170 | 0.176 |
| C4 | 0.142 | 0.220 | 0.114 | -0.059 | 0.234 | 0.209 | 0.070 | 0.175 | 0.161 |
| MMN latency |  |  |  |  |  |  |  |  |  |
| Fz | -0.125 | -0.087 | -0.167 | -0.191 | 0.076 | -0.087 | -0.092 | -0.073 | 0.000 |
| Cz | -0.142 | -0.094 | -0.185 | -0.218 | 0.056 | -0.080 | -0.126 | -0.087 | -0.020 |
| Pz | -0.103 | -0.086 | -0.188 | -0.138 | 0.083 | -0.073 | -0.105 | -0.070 | 0.014 |
| C3 | -0.153 | -0.111 | -0.223 | -0.201 | 0.081 | -0.120 | -0.111 | -0.107 | -0.025 |
| C4 | -0.135 | -0.094 | -0.191 | -0.187 | 0.113 | -0.112 | -0.074 | -0.079 | -0.003 |
| **Notes:** **P<*0.05. Spearman’s correlation coefficients (with Bonferroni-adjusted *P*-values) were obtained separately for CATS scores and CAARS scores.  **Abbreviations:** ASD, autism spectrum disorder; CAARS, the Conners’ Adult ADHD Rating Scale; TSR, CAARS total score of raw scores; IMP, inattention/memory problems; HR, hyperactivity/restlessness; IEL, impulsivity/emotional lability; PSC, problems with self-concept; AI, ADHD Index; ASI, *DSM-Ⅳ* ADHD symptom inattentive Symptoms; ASHI, *DSM-Ⅳ* ADHD symptom hyperactive/Impulsive Symptoms; AST, *DSM-Ⅳ* ADHD symptom ADHD symptoms total. | | | | | | | | | |
