# Supplementary material for: Associations of childhood experiences with event-related potentials in adults with autism spectrum disorder
Source: Sci Rep. 2020 Aug 10;10:13447. doi: 10.1038/s41598-020-70409-z (PMC7417533; doi:10.1038/s41598-020-70409-z)
Supplement: Supplementary file 7 — Supplementary Table 3. [file 41598_2020_70409_MOESM7_ESM.docx]

| **eTable 3** Correlations between CATS scores and CAARS scores | | | | | | | | | |
| --- | --- | --- | --- | --- | --- | --- | --- | --- | --- |
|  | Spearman’s correlations | | | | | | | | |
|  | **CAARS scores** | | | | | | | | |
|  | TSR | IMP | HR | IEL | PSC | AI | AIS | AHIS | AST |
| **CATS scores**  **Control** |  |  |  |  |  |  |  |  |  |
| CTS | 0.349 | 0.249 | 0.265 | 0.278 | 0.341 | 0.154 | 0.212 | 0.279 | 0.371 |
| SA | NA | NA | NA | NA | NA | NA | NA | NA | NA |
| Punishment | 0.149 | 0.083 | 0.073 | 0.145 | 0.456 | 0.142 | -0.107 | 0.045 | 0.269 |
| NNHA | -0.016 | 0.011 | 0.197 | 0.058 | 0.018 | -0.308 | 0.214 | -0.059 | 0.020 |
| EA | 0.488 | 0.431 | 0.328 | 0.153 | 0.352 | 0.345 | 0.307 | 0.471 | 0.495 |
| Other | 0.376 | 0.384 | 0.030 | 0.225 | 0.185 | 0.341 | 0.045 | 0.319 | 0.527 |
| **ASD** |  |  |  |  |  |  |  |  |  |
| CTS | 0.738* | 0.700* | 0.726* | 0.627* | 0.708* | 0.667* | 0.722* | 0.737* | 0.746* |
| SA | 0.447 | 0.394 | 0.551 | 0.379 | 0.649* | 0.373 | 0.544 | 0.487 | 0.622* |
| Punishment | 0.483 | 0.390 | 0.418 | 0.486 | 0.139 | 0.409 | 0.392 | 0.407 | 0.443 |
| NNHA | 0.691* | 0.674* | 0.679* | 0.587* | 0.693* | 0.631* | 0.674* | 0.691* | 0.700* |
| EA | 0.570 | 0.585* | 0.612* | 0.412 | 0.520 | 0.572 | 0.599* | 0.629* | 0.566 |
| Other | 0.604* | 0.502 | 0.603* | 0.599* | 0.587* | 0.543 | 0.702* | 0.619* | 0.678* |
| **Notes:** **P<*0.05. Spearman’s correlation coefficients (with Bonferroni-adjusted *P*-values) were obtained separately for CATS scores and CAARS scores.  **Abbreviations:** ASD, autism spectrum disorder; CATS, the Japanese version of the Child Abuse and Trauma Scale; CTS, CATS total score; SA, sexual abuse; NNHA, neglect/negative home atmosphere; EA, emotional abuse; CAARS, the Conners’ Adult ADHD Rating Scale; TSR, CAARS total score of raw scores; IMP, inattention/memory problems; HR, hyperactivity/restlessness; IEL, impulsivity/emotional lability; PSC, problems with self-concept; AI, ADHD Index; ASI, *DSM-Ⅳ* ADHD symptom inattentive Symptoms; ASHI, *DSM-Ⅳ* ADHD symptom hyperactive/Impulsive Symptoms; AST, *DSM-Ⅳ* ADHD symptom ADHD symptoms total; NA, non applicable. | | | | | | | | | |
